# An Expanded Framework for Situation Control

**DOI:** 10.3389/fnsys.2022.796100

**Published:** 2022-07-28

**Authors:** James Llinas, Raj Malhotra

**Affiliations:** ^1^Industrial and Systems Engineering Department, University at Buffalo, Buffalo, NY, United States; ^2^U.S. Air Force Research Laboratory Sensors Directorate, Wright-Patterson Air Force Base, Dayton, OH, United States

**Keywords:** stochastic control and time-varying systems, situation control, situation assessment, estimation, prediction

## Abstract

There is an extensive body of literature on the topic of estimating situational states, in applications ranging from cyber-defense to military operations to traffic situations and autonomous cars. In the military/defense/intelligence literature, situation assessment seems to be the *sine qua non* for any research on surveillance and reconnaissance, command and control, and intelligence analysis. Virtually all of this work focuses on assessing the situation-at-the-moment; many if not most of the estimation techniques are based on Data and Information Fusion (DIF) approaches, with some recent schemes employing Artificial Intelligence (AI) and Machine Learning (ML) methods. But estimating and recognizing situational conditions is most often couched in a decision-making, action-taking context, implying that actions may be needed so that certain goal situations will be reached as a result of such actions, or at least that progress toward such goal states will be made. This context thus frames the estimation of situational states in the larger context of a control-loop, with a need to understand the temporal evolution of situational states, not just a snapshot at a given time. Estimating situational dynamics requires the important functions of situation recognition, situation prediction, and situation understanding that are also central to such an integrated estimation + action-taking architecture. The varied processes for all of these combined capabilities lie in a closed-loop “situation control” framework, where the core operations of a stochastic control process involve situation recognition—learning—prediction—situation “error” assessment—and action taking to move the situation to a goal state. We propose several additional functionalities for this closed-loop control process in relation to some prior work on this topic, to include remarks on the integration of control-theoretic principles. Expanded remarks are also made on the state of the art of the schemas and computational technologies for situation recognition, prediction and understanding, as well as the roles for human intelligence in this larger framework.

## Introduction and Review of Current Research

**FIGURE 1:**
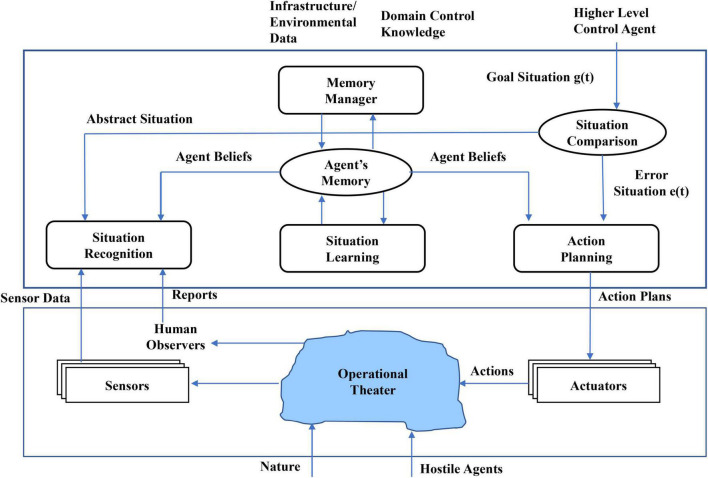
Concept of cognitive situational control derived from Jakobson (2017).

The concept of a “situation” can be thought of as describing a portion of a real-world that is of interest to a participant in that portion of the world. An understanding of a situation is needed and useful toward guiding or assessing the need for possible action of the participant in that situation. Action of a participant may also be needed to possibly alter the situation if it is in an undesirable state (assuming resources capable of affecting the situation are available), or for the participant to alter his position in the situation. For a human participant, the mental faculties of human cognition, such as consciousness (awareness), reasoning, formation of beliefs, memory, adaptation, and learning, frame the functional aspects of a process of cognitive situational understanding, related to the notion of sensemaking (see, e.g., Pirolli and Card, 2005; Klein et al., 2007).^1^ Acting on the situation, however, leads to the process of cognitive situation control, as well described in various of Jakobson’s papers (Jakobson et al., 2006, 2007, Jakobson, 2008; Jakobson, 2017) that, in part, motivated this work. A depiction of that process is shown in Figure 1 taken from Jakobson (2017); we offer here an abbreviated description of that process. The cycle starts with the existence of some (real, true) condition in the world, shown here by Jakobson as the “Operational Theater” which, as shown, can be affected by nature (that is, a context affected/defined by various contextual factors) and possibly of hostile or adversarial agents. That real situation is observed by imperfect and often multiple, multimodal sensors, and possibly human observers to support an estimation process that yields a “recognition” of the situation (a state estimate) that may be reasoned over by a human agent, or that provides an input to a subsequent automated process. (The situational picture so derived is understood to be only a part of some larger situational construct.) Jakobson calls this estimate the “Abstract Situation” in Figure 1. Given the current, recognized situation derived largely from observation, a Situation Learning process evolves from what we will call a contextual learning or a model-building process that could also be called Situation Understanding. Such a process implies an ability to develop a generalized, broader conception from the particulars of the current recognized picture and exploiting contextual factors either known *a priori* or collected in real-time. This process is similar to Bruner’s view that “mental modeling is a form of information production inside the neuronal system extending the reach of human cognition ‘beyond the information given”’ (Bruner, 1973). Following Jakobson, the recognized, learned situation is compared to a goal situation that presumably can be specified *a priori* or in real-time, and a difference is computed between the two situational states by a Situation Comparator function. That difference can be considered, from a control process point of view, as an “error” signal; if that (likely stochastic) difference is high enough (in consideration of an estimated state variance), actions need to be contemplated and assessed in a decision-making process, and once defined are enabled onto the current situation in an effort to “move” the situation toward the goal state. Note that Actions or Effects on to the situation can only be realized through whatever “Actuators” or Resources may be available to this control process.

There are two classes of ‘‘Resources’’ in this characterization: Observational Resources and ‘‘Actuators’’ or Resources that can enable changes in the real situation; these could also be called ‘‘Effectors.’’ The effective design of a process of managing these resources raises some challenges. For the Observational Resources, they first of all have to support the process that forms a recognized situational picture, possibly in the face of the ‘‘Five V’s’’ of modern-day Big Data environments,^2^ since this process does not start without an (estimated) recognized situational picture. To the extent then that the Observational Resources are a fixed resource set, and have any slack in their employment, they can also be used/multiplexed to support the employment of Effectors, as Effectors will need to be directed in some way. We submit that there is a time delay of possibly widely varying extent between the time of (initial) Situation Recognition and the eventual time of action of the Effectors; that is, most situations are continually unfolding and changing; they are dynamic. This being the case, it can be that there is a meaningful difference between the initial recognized situation and the situation that is eventually acted upon; such differences may result in very incorrect results of Actions if not accounted for. Thus, we assert that there will usually be a need for a Situation Prediction capability to create a temporal synchronization in this control process by propagating the situational estimate to the (expected, estimated) time of action. Then, just before acting, the predicted situation should be verified, this also requiring Observational resources. In sum, the Observational Resources will be shared over three different functional operations, as follows:

• Synchronizing Observation to Situational Velocity, Volume, Variety, Veracity, and Value in support of Situation Recognition
• Observation Multiplexing to support employment of Resources/Effectors
• Observation Multiplexing to support Situation Prediction confirmation.

A factor that will be very important in determining the process context for Situation Management and Control is the assessed rate at which the situation is unfolding; that is, the Operational Tempo (“OpTempo”) of the situation. This factor needs to be weighed in relation to both the scanning/sampling rate of the Observational Resources, the prediction interval, sensor resolution factors, and in fact the viability of the overall process; if the situation is unfolding at a rate faster than it can be feasibly observed, forming dependable situation estimates will be very difficult, and situational predictions will be equally hard. This balance changes the dependence of the Learning/Understanding process between *a priori* knowledge and real-time observational data; uncertainties in the consequent estimated situation will also be affected. Estimating situational OpTempo should therefore be a fundamental requirement of the Situation Recognition function, as it is a critical process design and management parameter, setting the overall “clock” for this control process. The notion of OpTempo is in the fashion of a meta-metric, since any situation will be comprised of multiple component processes unfolding at varying rates. Note too that there are optimization issues lurking here, as regards defining how optimal co-employment of bounded Observational Resources will be managed across these process needs.

Jakobson does not elaborate on the functions of Situation Learning nor on the Memory-based processes shown in Figure 1 (by choice, deferring those topics to future publications). He does elaborate on the functions of Situation Recognition as a tree-like hierarchical structure of component situation recognition sub-processes. A disaster-based use case is described within which an action-taking process that is also layered is elaborated. Jakobson, along with others on various occasions, has produced a number of papers on the central themes of cognitive situation management and many related topics that bear on the overarching topic of situation management (see prior citations and Jakobson et al., 2006; Jakobson, 2008).

To provide a historical perspective related to the process of situation control, we cite here the work of John Boyd, a military strategist and United States Air Force Colonel who in the 1980’s put forward the paradigm that has come to be called the “OODA Loop,” OODA an acronym for Observe-Orient-Decide-Act (see Boyd, 1986), but there are many papers, and a wide range of publications related to this paradigm if one searches on the web. It should be clear that these functions are quite similar to those depicted in Figure 1, with “Orient” perhaps needing clarification. Before remarking on Orient, it is emphasized that the OODA process was framed as a mental process, and then was studied by many to expand the framework to a potentially computational basis. Orient then was about mental modeling that built a mental model of a situation by consideration of prior knowledge (long-term memory), new information, cultural factors (a contextual effect), and other factors. This situation control type paradigm has found its way into business intelligence settings, game theory, law enforcement, and a multiplicity of other applications. A thorough review of the OODA process is provided in Richards (2012), although there are many publications about this process that addresses situation control.

Our intent in this paper is to expand the framework of cognitive control in terms of our views of several other component processes (forthcoming), and in discussing these additional processes, to relate them to research and capabilities in the cognitive neurosciences and machine understanding domains.

## Situation Control in Crisis Management

There is a large literature on crisis management and disaster management. In many cases, the characterization of the process begins with an assumption that certain of these problems can be anticipated, since in many cases an assessment of vulnerability to specific types of crises can be analyzed, such as in the cases of natural disasters. The ability to achieve Situation Recognition in these cases benefits from recognizing *anticipated* early signals of the onset of the event, among other factors. However, there are many other crises that do not follow this model, either because they are of a rare type or perhaps because they are perpetrated by some actors; situation recognition in these cases is both more difficult and will also take more time for evidence accrual. Perpetrated crises are analogous to military-type crises and can have similar properties such as the employment of deception techniques and other complications; these factors re-orient the situation assessment process to one of adversarial reasoning. In any setting involving situation state estimation, an early question has to do with whether the setting is a natural one where phenomena are driven by natural causes or whether the setting comprises a two-sided, adversarial context. The case involving adversaries can be related to the case of “Information Warfare,” (IW), where the two sides are manipulating information, the bases for perception and inference, to their advantage. The larger purpose of these operations is to manage adversarial perceptions by structuring the information available to an adversary to be compliant with that perceptual construct. Another topic related to deception is denial of information by covertness, camouflage, jamming, and other means. Deception and denial strategies work because of exploitation of reasoning errors, cognitive limitations, and cognitive biases (Elsaesser and Stech, 2007). The most important errors are:

• Reasoning causally
• Failure to include a deception hypothesis
• Biased estimates of probabilities
• Failure to consider false positive rates of evidence.

In our own experience in dealing with an earthquake disaster case, there was the additional complication of multi-jurisdictional participants, all taking different views of the integrated situation and what resources are to be deployed and controlled. This latter case involved additional processes of consensus-forming and complex communications to both recognize and predict situational states. In our disaster example and in most crisis problem contexts, a top priority is life-saving and casualty recovery, and the situation to both understand and control is that which relates to all of the dimensions of casualty-recovery operations. Such operations are dependent on vulnerable infrastructure components such as airports, ambulance depots, and electrical power. In addition, it is very typical in crises that there are cascading effects; in the case say of an earthquake, the tremors will cause primary problems such as building collapses but will in addition rupture gas lines leading to fires as secondary threats. These same cascading events occur in other crises as well, such as in wildfires, where entry and exit routes are compromised by evolving fire patterns, and where wildfire observation such as from drones is affected by dense smoke patterns; all of these factors drive a need to model the dynamics of situation control patterns. A core challenge in all situation control problems is achieving synchronization of the situation recognition, prediction, and understanding processes with control-related and action-taking processes. That is, there are the issues of gaining situation awareness and maintaining situation awareness, while comparing situational conditions to those desired and subsequently deciding on specific control actions.

### Related Work; A Sampling

As noted above, there is a lot of literature on crisis and disaster management for which the topic of situation control would seem to be of interest. Relatively few papers in this field, however, address end-to-end process issues and models in the systemic context of this paper, although there many papers that address portions of the entire process; we sample a few here.

For example, it is clear that any Situation Control process must also be managing data and might require ancillary analytical support operations. The paper by Hristidis et al. (2010) provides one overview of data management and analysis processes in a stressing disaster-type situation. Information extraction, retrieval, and filtering processes (similar to data preparation processes in data fusion operations) are needed to extract relevant data of satisfactory quality for subsequent operations. Aspects of the supporting process infrastructure are addressed here as well, such as the need for a consistently formatted data base. This work is focused on textual data (often called “soft” data to distinguish it from quantitative “hard” data from electromechanical sensors), an important class of data for situation control, often not addressed.

Zambrano et al. (2017) provide an interesting aspect of a modern-day situation control problem regarding the use of cellphones; most modern contexts where a situation is evolving will involve cellphones carried by many people, and cellphone data of various type can contribute to both the estimation of the situation and aid in controlling the situation. This paper interestingly brings together a detailed data fusion process model, following the well-known JDL Data Fusion Process Model (see Llinas et al., 2004) and builds an end-to-end situation estimation process model based on cellphone-captured data. The main contribution here is the messaging protocol for information exchange in complex cellphone networks, and support to early warning notifications in real time.

The paper by Van de Walle et al. (2016) provides some interesting views for enriching raw incoming information by adding a summary of the information received, and by channeling all incoming information to a central coordinator who then decides upon further distribution within the team. This paper is largely about information quality, a factor that is important in all information operations. In a manner similar to assigning “pedigree” to information sources based on analytical or experiential bases, this paper discusses notions of information richness that can be based on reputation or on analytical methods that compute metrics for information sources based on notions of completeness or timeliness, and other such quality-influencing factors. While information can be enriched in various ways, in this research “enriched information” is defined as information that combines information from different sources and is represented in a format with which professional crisis responders are familiar, similar to the association and combining operations in a data fusion process. Information that is not aggregated nor represented in a specific format is considered “raw” or non-enriched. This work carries out a series of experiments to explore the hypotheses related to information enrichment and centralized decision-making, concluding that that enriched and non-enriched information conditions are significantly different only if information is centralized.

In Costa et al. (2012), a Situation Modeling Language (SML) is developed, which is a graphical language for situation modeling, and an approach to situation detection and recognition based on the SML model is realized by linking the model to a rule-based scheme. The motivation for this paper comes in part from a view of Kokar et al. (2009) that argues, from an ontological point of view, that “to make use of situation awareness […] one must be able to recognize situations, […] associate various properties with particular situations, and communicate descriptions of situations to others.” In addition to supporting an ontological foundation related to anything having to do with situations, this paper has many features that resonate with our own ideas, for example in defining situations as composite entities whose constituents are other entities, their properties, and the relations in which they are involved. This leads to an approach which is similar to an ontological approach that we also argue for in this paper, and also to a graphical construct that we also support as the correct modeling basis for these problems. This work also concerns itself with formal semantics which are quite necessary for these problems since clear semantics aid in clarifying combinatoric complexities of layered situational constructs. Previous work by Dockhorn et al. (2007) addresses what could be called the context of situation development, where an “invariant” is defined as the necessary and sufficient conditions for a situation to exist. Addressing context and its importance in situation estimation is also addressed from various points of view in works by Snidaro et al. (2016). Yet other work of Costa et al. (2006) addresses a distributed rule-based approach for situation detection. When well-designed and developed, rule-based systems can be both efficient to develop and to effective to employ, but there are many lessons-learned and limitations of rule-based systems that need to be considered (e.g., Nazareth, 1989), such as scalability, blindness to data not included in the rules, and coverage of unbounded parameter values. While the foundational and systemic aspects of these works are very relevant to our discussion on situation control, the authors point out in more than one of these papers that evaluations of these prototype implementations are under development.

All of these works are focused on the estimation function and associated processes for developing capabilities to estimate situational states. Collectively, many systems engineering issues are addressed, to include data management, ontological issues, modeling of situations, and other related functions for situational estimation. In that regard, they are solid research projects but they are not directed to the holistic, closed-loop situation control process that involves decision-making once situational states are determined.

## Proposed Functional Expansion of the Baseline Framework: Overview

While Jakobson provides a sound initial foundation for a process description of situation control, we suggest various enhancements of this process description. A first remark is that the level of specificity of the meaning and construct of a “situation” needs to be elaborated; we see this ideally as resulting from a formal ontological development. At the highest level of abstraction, one could say that a situation is a set of entities (here, writ large, meaning not only physical entities and objects but events and behaviors) connected by a set of relations. Relations bring a new challenge to observation-based estimation because relations are not observable by conventional sensing devices, sometimes called “hard” sensors, meaning electromechanical type devices such as radars and imaging systems. Hard data produces features and attributes of entities in the situation from which inter-entity relations could be reasoned. It is possible that “soft” data such as social media data may apply to a situation control problem, in which case such data may, if based on human observation, reasoning, and judgment, yield direct estimates of inter-entity relations. Contextual type data, that imputes influences on the estimation of entities and relations, would also be fused in a robust observation and data fusion process to aid situation estimation processes. Along with the entity ontology, a relation ontology is also needed so that the specifics of a labeled, specific situational state can be assembled from these components. That assembly requires a higher level of abstraction in inferencing. Thus, Jakobson’s situation recognition process will need to be supported by an ontological foundation where entities, relations, and labeled situational states are coupled to the fusion and recognition processes that will have to assemble the recognized, labeled situational state by exploiting this framework, and also by accounting for the various uncertainties in the integrated observational and inferential processes.

Another suggestion relates to the need for accounting for time. As we remarked previously, the real world is always dynamic, and so situations are in a constant process of unfolding; situations can be labeled as continuously valued random variables. Thus, we assert the need for a Situation Prediction process that is the means for maintaining situation understanding over time. How such a process may be framed depends on how the situation state is modeled; for example, a situation could be represented as a graph (entities as nodes, relations as arcs) or as a pattern of variables in the form of a time-series, or yet other representational forms. Many strategies for prediction address the problem as a pseudo-extrapolation of some type, projecting the most likely evolution of the dynamic sub-parts of a current situation. This brings in the need for Situation Understanding, which we characterize as a process that enables generalization from the particulars of the moment. Situation Understanding admits to adding knowledge and thus adding (or subtracting) new piece-parts of the situational construct, thus enabling more insightful projection of estimated situation dynamics. At some point in time or as part of an ongoing process, an assessment of whether the situation is satisfactory or not is typically carried out; this requires a specification of some desired situational state (as previously noted, Jakobson calls it a Goal Situation in Figure 1) that is the basis for comparison. Executing this step thus requires a process for Situational Comparison. However, executed, the comparison process yields what could be called an “error signal” as would exist in any control process, as Jakobson points out; we assert that this error signal will have stochastic properties, since the estimated situational state, and perhaps the goal state as well, will have stochastic-type error factors embedded in the calculations. The error signal requires assessment as to whether any action is required, and so there is a question as to “degree” of error, and if the error is stochastic, issues of variance in this error variable will factor into the severity assessment.

**FIGURE 2:**
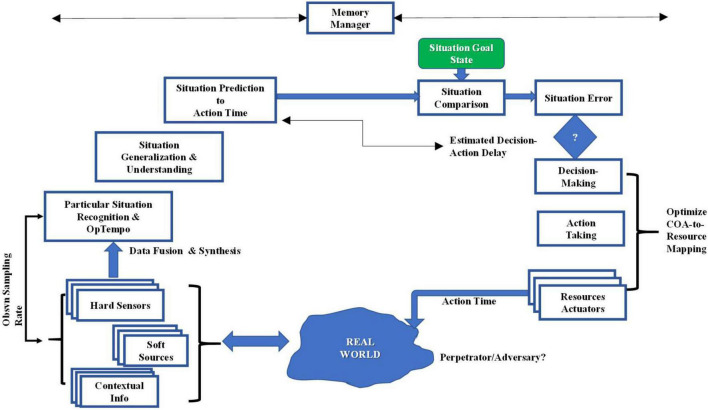
Expanded framework of cognitive situation control.

Another timing issue also arises at this point: this relates to the issue of synchronizing the action-taking and the situation prediction processes in order that the planned action is in fact acting on the intended world situation at the action-time. All these processes consume time, and an estimate of the sum of the decision-time and acting-time will set a requirement for situation prediction so that the actions that occur are acting onto the expected situation at that time; thus, there are process interdependencies (see Llinas, 2014) for further remarks on this point). These expanded remarks and functional needs are depicted in Figure 2 that shows our suggestions for an expanded framework of situation control:

## Expanded Functional Review: State of the Art and Challenges

### Situation Recognition

One definition of “recognize” is to “perceive something previously known,” implying that a model-comparison type process is employed for recognition. But even before a model is conceptualized, a modeling framework is required to set a norm for the structure and content of such a model; this requirement brings into our discussion the need for a situation ontology. To our knowledge, no fully and well-developed, formalized ontological specification of a situational state exists that has been taken up broadly by researchers addressing the kind of problems we are discussing here (e.g., the data fusion community). There has been a fairly large number of publications that offer representational schemes for situations, some labeled as ontologically-based, but those models have not been broadly applied (see Dousson et al., 1993; Boury-Brisset, 2003; Baumgartner and Retschitzegger, 2006; Little and Rogova, 2009; Cardell-Oliver and Liu, 2010; Almeida et al., 2018, that are just a sampling). As situations are rather complex world states, processes trying to estimate these states need to take a position on what the components of situations are, as most approaches can be labeled as bottom-up, assembling situational state estimates from estimates of the components. Development of a rigorous situational ontology and harmonization of its use across a community is a very complex matter. It would seem that such an issue should fall to the portion of a community addressing its engineering methods, and the regularization of top-down system engineering approaches; how this issue will unfold going forward remains unclear.

As it is clear that situations evolve and change over time, we need to think about the tempo of situation recognition as a process, e.g., as a “freeze-frame” depiction or perhaps an interval-based depiction. This issue muddies the distinction between recognizing a situation and prediction and updating of situations; the underlying issue is that a situational state is a continuous variable, an emphasis previously pointed out. Importantly, perhaps even crucially, the ability to assess the situation evolution rate/OpTempo is needed to specify the required observational rates of situation components, in the fashion of a “Nyquist” criterion for signal sampling. Clearly if the observational rates across the sensor suite are not tailored to the situational tempo, the entire situation estimation and control framework is compromised.

#### Sampling of Computational Methods for Situation Recognition

In Mannila and Ronkainen (1997), as in other works reviewed here, a situation is depicted as a series of events, i.e., an event sequence. For many papers, as we will see, the issue of comparison evolves around developing notions of similarity. In Mannila and Ronkainen (1997) then, there is the issue of defining similarity across event sequences. Building on the intuition that differences or similarities in sequences relates to how much work has to be done to convert one sequence to another, they define an “edit distance” measure of similarity. These edit distance measures are computed using a dynamic programming approach. Sequence transformation operations such as insert, delete, and move are formed, as well as a cost measure. From this framework, an optimization function can be developed to compute the minimum cost of a sequence edit between sequence pairs. Some limited empirical results are developed that show reasonable performance of this exploratory approach. Sidenbladh et al. (2005) propose an approach based on using random sets as the representational form for situations. This paper compares rolling situation predictions as a use case where the situation predictions at two different times are normalized due to estimation noise differences, arguing that prediction error is proportional to prediction time. Given that normalization, they define a standard norm as a similarity/difference measure and also point out that the Kullback-Liebler measure^4^ is inappropriate for this purpose. In some of our own work, we have depicted situations as graphs, following a simple situation definition, as previously mentioned, as a set of entities connected by a set of relations. Situation similarity then can be assessed by any of the many existing types of metrics for graph comparison (see e.g., Hernandez and Van Mieghem, 2011). Which metrics are best will depend on the graph details; for example, relations among entities can be directed, and so comparison would then require metrics that account for directed arcs in the representational graphs for the situations being compared. There are metrics that can compare both the global and local characteristics of two graphs; methods of this type have been used for anomaly detection in situational analysis. Since the description of any situational state will employ language to label the situational components (entities) and their relations, notions of situational similarity may also involve issues of semantic similarity in the terms employed. Our research in hard and soft data fusion for disaster response needed to address this issue, which has been studied extensively, since semantic similarity and whether words mean the same thing is a core issue in many application settings. A hierarchically structured ontology or taxonomy can be useful in estimating the semantic similarity between nodes in the taxonomic network. Two specific approaches used to determine the conceptual similarity of two terms in this type of network are known as node and edge-based approaches. The node-based approach relates to the information content approach while the edge-based approach corresponds to the conceptual distance approach. The edge-counting measures are based on a simplified version of spreading activation theory (Cohen and Kjeldsen, 1987) that asserts that the hierarchy of concepts in an ontology is organized along the lines of semantic similarity. Thus, the more similar two concepts are, the more links there are between the concepts, and the more closely related they are Rada et al. (1989). The node-based measures are based on the argument that the more information two terms share in common, the more similar they are, and the information shared by two terms is indicated by the information content of the terms that subsume them in the taxonomy. Data association methods employed in data fusion have been used to assess whether two situation states have the same objects in them (e.g., Stubberud and Kramer, 2005); these metrics used ideas from metric spaces and cardinality principles to compute object-set similarities. Other techniques for assessing situational similarity can be drawn from measures for assessing similarity of sets such as the Jaccard Similarity and the Overlap Coefficient (Rees, 2019).^5^ Similarity of relations is also of interest, and the methods of ontological similarity could be used for relation-labels as well as methods from Fuzzy Logic and latent variable type analyses (Turney, 2006). Finally, (Gorodetsky et al., 2005) develop a situation updating method that addresses the issue of asynchronous data with a data ageing scheme, the missing data issue with a direct data mining approach, and a situational state classification scheme based on a rule-based approach, in an effort to account for these various aspects of situation updating in an integrated approach.

### Situation Prediction

As noted in section “Proposed Functional Expansion of the Baseline Framework: Overview,” the requirement for a situation prediction (SP) process is linked to the time of action onto the predicted situation. As for most prediction, projection, or extrapolation processes, the difficulty and accuracy of such processes is linked to the temporal degree of projection (how far ahead) and the rate of observation and input of any data that the projections depend on; this is not just sensor/observational data but contextual and soft data as well. We have emphasized the importance of the temporal aspects and the need to maintain situation awareness; that emphasis is acknowledged in various recent publications (e.g., Blasch, 2006; Niklasson et al., 2008; Baumgartner et al., 2010; Foo and Ng, 2013). Research areas where situation prediction has been addressed include cyber defense, for attack/intent projection, autonomous vehicles where traffic situation prediction is crucial, and also crisis/disaster management, to guide response services.

#### Sampling of Computational Frameworks for Situation Prediction

Two application areas where SP is addressed are those related to Cyber SP for cyber-defense and Traffic Situation SP related to autonomous car systems. A broad area where SP has also been addressed is in a wide variety of game settings, from Chess to Wargaming to Video Gaming. Most game environments, however, have various rules that can constrain the evolution of situations and thus provide a constrained framework within which to explore SP, although many other settings will also have constraints. We choose to show the SP framework of Baumgartner et al. (2010) for traffic prediction that describes a holistic approach that shows the joint exploitation of an SA Ontology and, in this case, Colored Petri Nets (CPN) as an SP estimation/modeling scheme.

In the traffic/autonomous car application, it is desired to predict critical situations from spatio-temporal relations between objects. These and other relations can be expressed by employing relation calculi, each of them focusing on a certain spatio-temporal relation, such as mereotopology-based (“part-of” based), spatial orientation, or direction. According to Baumgartner et al. (2010) these calculi are often modeled by means of Conceptual Neighborhood Graphs (CNGs, see Freksa, 1991); as noted in this paper, the CNG’s impose constraints on the existence of transitions between relations, thus providing a way to bound the complexity of relation modeling. CNGs can be used for modeling continuously varying processes, and have been used in a variety of related applications. Representing CNGs as CPNs can lead to increasing prediction precision by using precise ontological knowledge of object characteristics (if the ontology is done well) and interdependencies between spatio-temporal relations. This can lead to increased prediction explicitness in their approach by associating transitions with dynamically derived distances for multiple view-points. These so-called Situation Prediction Nets (SPN) in Baumgartner et al. (2010) are derived automatically from the available situation awareness ontologies. The research described in this paper is among the few that proactively integrate an ontological framework for relations and situation structures with a computational strategy for SP.

In Salfinger et al. (2013), a situation’s evolution is modeled as a sequence of object-relational states it has evolved through, i.e., the sequence of its situation states. This approach discretizes the continuous evolution of the monitored real-world objects into a sequence of their different joint relational states defined by various relations between those objects, defined in an “alphabet” or what could be called a bounded ontology. Thus, the problem of predicting a monitored situation’s evolution is cast as a sequence prediction problem. This technique is also applied here to the traffic-situation prediction problem. This approach employs a Discrete Time Markov Chain scheme; this is preceded by a situation-mining analysis to define the situation state-space “alphabet,” learned from human-labeled state sequences.

As previously remarked, works on SP can also be found in the cyber-defense domain. In Husák et al. (2019), a survey of such methods is provided. Their approach addresses four categories of predicative capability. The first two of these categories are attack projection and intention recognition, in which there is a need to predict the next move or the intentions of the attacker, third is intrusion prediction, in which predictions are developed of upcoming cyber-attacks, and fourth is network security situation forecasting, in which projections are made of the cybersecurity situation in the whole network. Across these applications, the paper reviews two broad categories of prediction techniques: discrete-time approaches, and continuous-time approaches. The discrete-time techniques include: “attack graphs” that probabilistically model initial and successor states of a postulated attack process. As in Salfinger et al. (2013), the state-space if often defined by a data mining analysis. The predictions using attack graphs are based on traversing the graph from an initial state and searching for a successful or most-probable attack path. A number of papers are cited in the survey that employ variations of this technique. Bayesian Nets and Markov techniques, as well as Game-theoretic methods are among the other discrete-time approaches reviewed. The continuous-time methods reviewed fell in to two categories, time-series methods and “gray” methods. These methods were largely used for whole-network predictions involving forecasts of the numbers, volumes, and composition of attacks in the network and their distribution in time.

#### Some Views From Cognitive Neuroscience

**FIGURE 3:**
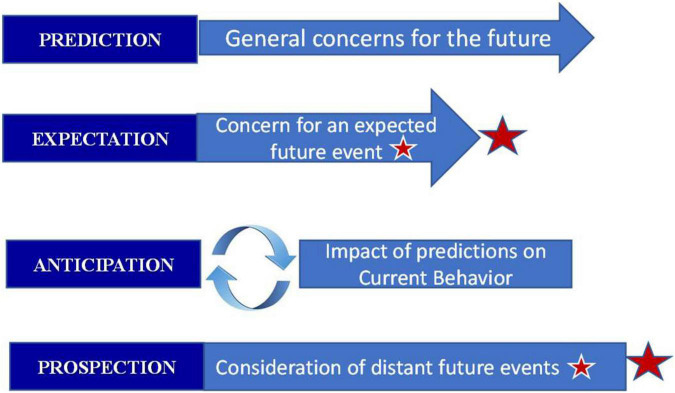
Distinctions in prediction-anticipation-prospection derived from Bubic et al. (2010).

We have maintained that Situation Prediction is a functional requirement in the process of Situation Control. There are relatively few frameworks offered in the technical engineering literature for Situation Prediction (as just discussed) but there are also some paradigms for this process in the computational neuroscience literature. For example, Bubic et al. (2010) provide one overview of such processes in the brain. In this paper, distinctions are made in relation to the horizon over which predictions might be made (as we have also mentioned previously). For example, the term “expectation” is said to reflect the information regarding the spatial and temporal characteristics of an expected event, whereas “anticipation” describes the impact of predictions on current behavior, e.g., decisions and actions based on such predictions, and “prospection” is described as an ability to “pre-experience the future by simulating it in our minds.” These distinctions are shown in Figure 3.

**FIGURE 4:**
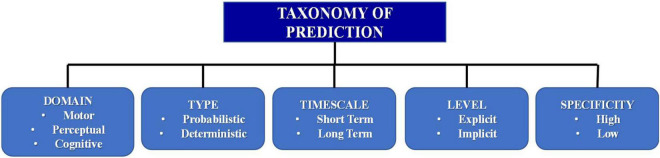
Factors influencing the nature of prediction derived from Bubic et al. (2010).

The main factors that influence the nature of a predictive process are characterized in Bubic et al. (2010) as shown in Figure 4.

Heeger, in a paper that provides somewhat detailed mathematical models of cortical processes (Heeger, 2017), suggests that prediction is one of three key cortical operations: (i) inference: where perception is a non-convex optimization that combines sensory input with prior expectation; (ii) exploration: here, inference relies on neural response variability to explore different possible interpretations; and (iii) prediction: inference includes making predictions over a hierarchy of timescales, not unlike suggested by Bubic et al. (2010) The starting point for this development is the hypothesis that neural responses minimize an energy function that represents a compromise between the feedforward drive and prior drive (drive ≈ neural signals). In these process models, the responses of the full population of neurons (across all channels and all layers) are asserted to converge to minimize a global optimization criterion, which Heeger calls an energy function. Specifically, the starting point for this model development is the hypothesis that neural responses minimize an energy function that represents a compromise between the feedforward drive and prior drive. Heeger says that predictive coding theories start with a generative model that describes how characteristics of the environment produce sensory inputs; Perception on the other hand is presumed to perform the inverse mapping, from sensory inputs to characteristics of the environment. Heeger’s approach suggests a different process for how the brain might predict over time, relying on a recursive computation similar to a Kalman filter, where the predictive basis functions serve the same role as the dynamical system model in a Kalman filter.

Returning to cognition, many researchers in the neuro and cognitive sciences have developed a view according to which prediction or anticipation represents a fundamental characteristic of brain functioning, suggesting that prediction is “at the core of cognition” (Pezzulo et al., 2007). Further, for many cognitive functions and neural systems, an ability to anticipate is a core requirement, such as in motor and visual processing and attention (Mehta and Schaal, 2002). According to Friston (2005), predictive processing is inherent to all levels of our organized neural system. It is suggested that predictions drive our perception, cognition, and behavior in trying to fulfill predictions by preferentially sampling features in the environment. Nevertheless, it can be expected that mismatches will occur, and the size of such mismatches (prediction error) creates a “surprise” that the brain tries to minimize in order to maintain present and future stability (Friston and Stephan, 2007). In reviewing Bubic’s paper, one comes away with the interpretation that anticipatory or predictive processing potentially reflects one of the core, fundamental principles of brain functioning which justifies the notion of “the predictive brain” seen in some papers.

These neuronal-level models are quite interesting in helping to understand how the brain develops predictions, but what is being predicted are anticipated human-based sensor signals that are important to human survival.

One such model, the Virtual Associative Network (VAN), is combined with active inference and presented elsewhere in this Frontiers special edition (Moran et al., 2021). This work presents a new, Cognitive-Partially Observable Markov Decision Process (C-POMDP) framework, extending the Partially Observable Markov Decision Process (POMDP) to account for an internal, cognitive model which attempts to contend with situation control considerations we outline here such as situation recognition, prediction, learning and understanding.

The C-POMDP framework presumes an active interaction between the agent and its environment wherein the agent interacts with the environment in repetitive cycles consisting of (i) sensing observable phenomena within the environment; (ii) estimating situational states, situation dynamics (behavior, op tempo, relations, etc.); (iii) predicting future states and rewards; and (iv) making decisions to maximize expected rewards. A key point here is that these estimation and decision-making processes are based upon an internal model which is maintained and updated by the agent as it reasons about experiences. In Moran et al. (2021), learning is facilitated by probabilistic reasoning and operations on a graph-based modeling structure which encapsulates associations between objects (entities, situation artifacts), behaviors, and relations.

### Situation Learning and Situation Understanding

The topics of learning and understanding have of course been extensively studied by a variety of research and application communities. These concepts have some relationship but they are also distinct from each other. Learning can be seen as dependent on (at least) two processes: observation and data gathering, and on experimentation and acting. Both processes produce real-time data that support inductive processes directed to gaining real-time knowledge. Understanding would seem to follow learning wherein the gained knowledge, along with archived knowledge, are exploited *in combination* to develop a *generalized* understanding of a world situation that allow development of a contextual perspective—a generalized perspective—of that situation. Generalization allows the recognition of the similarities in knowledge acquired in one circumstance, allowing for transfer of knowledge onto new situations. The knowledge to be transferred is often referred to as abstractions, because the learner abstracts a rule or pattern of characteristics from previous experiences with similar stimuli. Yufik (2018) defines understanding as a form of active inference in self-adaptive systems seeking to expand their inference domains while minimizing metabolic costs incurred in the expansions; the process thus also entails an optimization element directed at minimizing neuronal energy consumption. This view also sees understanding as an advanced adaptive mechanism in virtual associative networks involving self-directed construction of mental models establishing relations between domain entities. Understanding inter-entity relations is also a core element of situation understanding. Thus, the relationship between learning and understanding can be seen as complementary; understanding complements learning and serves to “overcome the inertia of learned behavior” when conditions are unfamiliar or deviate from those experienced in the past (Yufik, 2018). A challenge now receiving considerable attention with the new thrusts into AI is to understand how humans are able to generalize from very limited sampling. One approach fostered by Tenenbaum et al. (2011) and Lake et al. (2015) is based on probabilistic generative models, proposed as a basis for linking the psychological and physical aspects of the world. These techniques are being explored in DARPA’s Machine Commonsense program; however, these techniques will yield learning and understanding processes that create the foundational nuggets of what humans typically call ‘‘common sense’’ knowledge, often called tacit knowledge, and are far from a computational ability to understand situations of varying complexity. (An often-cited example of common, tacit knowledge that humans accrue is the learning of embedded rules of grammar that are learned over time from discrete sampling.) As most would agree that understanding involves uncertainty, whereas knowledge is often defined as ‘‘justified true belief’’ following Plato (yet acknowledging Gettier),^3^ it seems reasonable to explore probabilistic methods to model commonsense understanding. The issue of exactly how certain one must be about a belief to qualify as “knowing” has been called the boundary problem (Quine, 1987). We see that there are thus distinctions between understanding and knowledge; importantly, understanding can be possibly incorrect. Also important to this discussion, as just mentioned, is the process of generalization, a rather pervasive topic in psychology. In Austerweil et al. (2019), discuss the issue of learning how to generalize, which suggests that generalization requires postulating “overhypotheses” or constraints in effect on the hypothesis domain to be nominated. Some assert that such overhypotheses are innate but Austerweil et al. (2019) argue that they can be learned. In either case, the generalization framework is said to be Bayesian-based. Generalization has also been studied in Shepard (1987) that suggests an exponential metric distance between the stimuli as a basis to assert similarity, and in Kemp et al. (2006) that discusses the overhypotheses issue. We note that the issue of assessing similarity or degrees of association between disparate or multimodal data is broadly similar to the generalization question, and is a topic addressed in the field of multisensor data fusion. In those cases, techniques of multidimensional scaling, copulas, and manifolds have been used to develop scaling methods to relate such non-commensurate data.

### Situation Comparison

The assessment of any situation as to its acceptability or to the need for situation control and action-taking requires the specification of some basis for comparison; in Figure 1 Jakobson shows the Situation Comparator function needing a Goal Situation to be defined. As situational states can be rather complex, the bases of comparison could perhaps be done for portions of a situation rather than the entirety of a complicated, entangled set of situational elements. How any such comparisons would be done is also dependent on how one chooses to represent situations. Our search for literature related to this situation comparison issue shows that this issue has not been extensively addressed, and the methods proposed are of very different type, as described next.

#### Sampling of Computational Methods for Situation Comparison

In Mannila and Ronkainen (1997), as in other works reviewed here, a situation is depicted as a series of events, i.e., an event sequence. For many papers, as we will see, the issue of comparison evolves around developing notions of similarity. In Mannila and Ronkainen (1997) then, there is the issue of defining similarity across event sequences. Building on the intuition that differences or similarities in sequences relates to how much work has to be done to convert one sequence to another, they define an “edit distance” measure of similarity. These edit distance measures are computed using a dynamic programming approach. Sequence transformation operations such as insert, delete, and move are formed, as well as a cost measure. From this framework, an optimization function can be developed to compute the minimum cost of a sequence edit between sequence pairs. Some limited empirical results are developed that show reasonable performance of this exploratory approach. Sidenbladh et al. (2005) propose an approach based on using random sets as the representational form for situations. This paper compares rolling situation predictions as a use case where the situation predictions at two different times are normalized due to estimation noise differences, arguing that prediction error is proportional to prediction time. Given that normalization, they define a standard norm as a similarity/difference measure and also point out that the Kullback-Liebler measure^4^ is inappropriate for this purpose. In some of our own work, we have depicted situations as graphs, following a simple situation definition, as previously mentioned, as a set of entities connected by a set of relations. Situation similarity then can be assessed by any of the many existing types of metrics for graph comparison (see e.g., Hernandez and Van Mieghem, 2011). Which metrics are best will depend on the graph details; for example, relations among entities can be directed, and so comparison would then require metrics that account for directed arcs in the representational graphs for the situations being compared. There are metrics that can compare both the global and local characteristics of two graphs; methods of this type have been used for anomaly detection in situational analysis. Since the description of any situational state will employ language to label the situational components (entities) and their relations, notions of situational similarity may also involve issues of semantic similarity in the terms employed. Our research in hard and soft data fusion for disaster response needed to address this issue, which has been studied extensively, since semantic similarity and whether words mean the same thing is a core issue in many application settings. A hierarchically structured ontology or taxonomy can be useful in estimating the semantic similarity between nodes in the taxonomic network. Two specific approaches used to determine the conceptual similarity of two terms in this type of network are known as node and edge-based approaches. The node-based approach relates to the information content approach while the edge-based approach corresponds to the conceptual distance approach. The edge-counting measures are based on a simplified version of spreading activation theory (Cohen and Kjeldsen, 1987) that asserts that the hierarchy of concepts in an ontology is organized along the lines of semantic similarity. Thus, the more similar two concepts are, the more links there are between the concepts, and the more closely related they are Rada et al. (1989). The node-based measures are based on the argument that the more information two terms share in common, the more similar they are, and the information shared by two terms is indicated by the information content of the terms that subsume them in the taxonomy. Data association methods employed in data fusion have been used to assess whether two situation states have the same objects in them (e.g., Stubberud and Kramer, 2005); these metrics used ideas from metric spaces and cardinality principles to compute object-set similarities. Other techniques for assessing situational similarity can be drawn from measures for assessing similarity of sets such as the Jaccard Similarity and the Overlap Coefficient (Rees, 2019).^5^ Similarity of relations is also of interest, and the methods of ontological similarity could be used for relation-labels as well as methods from Fuzzy Logic and latent variable type analyses (Turney, 2006). Finally, (Gorodetsky et al., 2005) develop a situation updating method that addresses the issue of asynchronous data with a data ageing scheme, the missing data issue with a direct data mining approach, and a situational state classification scheme based on a rule-based approach, in an effort to account for these various aspects of situation updating in an integrated approach.

## Control Dynamics

We have described the overall control process so far as rather linear and feed-forward but there may be inter-functional interdependencies across each “situational” function described here. As multisensor data fusion processes are relevant information processes supporting situation control as candidate processes for situation estimation (see, e.g., Liggins et al., 2009) it is known that there can be inter-process dependencies that need to be addressed among data fusion, situation estimation, and decision-making processes (see Llinas et al., 2004; Llinas, 2014). In the case of data fusion processes, the approach to situation estimation is typically layered, following a “divide and conquer” approach typically employed for complex problems. The layered estimates are partitioned according to specificity, with lower levels estimating features of situational entities, and upper levels estimating aggregated multi-entity relational constructs. Thus, the layers share content about common entities that may be helpful to share in a synergistic scheme; for data fusion, Llinas et al. (2004) addresses some of the issues of this point. In the case where data fusion and decision-making processes are integrated in a single architecture, the inter-process dependencies exist because one process, data fusion, is estimating a situation and the other process is deciding about situations; these interdependencies are discussed in Llinas (2014). Further, the Action Planning and Action-Taking processes that depend on the possibly complex viable action-spaces of available resources (that is, the various situation-affecting actions that a given resource can execute) can lead to the need for an optimization-based approach to select the best resource to execute a particular situation-affecting action. Situation OpTempo and overall timing control again need to be considered since there can be delays in making the action-taking decisions (e.g., solving an optimization problem) and delays in employing a resource and realizing its intended effects. Consideration of these factors aids in estimating the time it takes to make a decision and the time for resources to act on the situation. An *a priori*/ongoing estimate of the sum of these times provides the time specification to the Situation Prediction function so that the system is predicting the situational state at the expected time of action from the resources; also discussed in Llinas (2014).

### Partially Observable Markov Decision Process

Control Theory offers a foundational problem formulation for many problems requiring Situation Control. Such problems presume an active interaction between an intelligent agent and its environment where:

• The agent exercises repetitive cycles of sensing the environment, executing actions and modifying them based upon feedback
• The agent seeks to maximize cumulative rewards received from the environment
• The agent iteratively maps an error signal into actions.

In the POMDP formulation, these problem elements are expressed as sets, and mappings between the sets. More specifically, the environment offers a set of states (S) and a set of rewards (R). The agent will iteratively draw from a set of observations (O), and choose from a set of actions (A). Here, the dynamics of the situation are characterized by a set of state transition probabilities (P), providing a mapping from a particular state at time *t* to a state at time *t* + *1* (P: s_t_→s_t+1_). The agent’s observations which are related to environmental state (S), are characterized by a set of observation probabilities (Z) which map state at time *t* to an observation at a time *t* or a later time *t* + *n* (Z: s_t_→o_t+n_, *n* = 0). Similarly, the relationship between rewards and underlying state received by the agent may be modeled deterministically or stochastically as related to state; If stochastic, the relationship between the states (S) and rewards (R) will be characterized by a reward probability mapping, Q (Q: s_t_→r_t+m_, *m ≥* 0). For further information on a POMDP modeling approach, the reader is referred to Bertsekas (1987).

Although the POMDP offers a principled problem formulation for complex situation control problems, it is well established that, for realistic problems, POMDP solutions often suffer from “the curse of combinatorial explosion” and approximate solutions methods are required for solution (Bertsekas, 1987). These approximate methods include, perhaps most notably, Reinforcement Learning methods (Spaan, 2012) which have been used extensively in some artificial intelligence solutions.

The authors contend that the POMDP offers a starting point for the control aspects of the situation control problem formulation but effective solutions for complex situation control problems will require that the relationships between pertinent situational factors governing state transition probabilities (P), observation probabilities (Z), and reward probabilities (Q) be understood. In practice, identifying the relevant situational factors and accurately modeling the relationships governing these mappings will be derived experientially, through situation learning as described in section “Situation Learning and Situation Understanding” above. Further, the temporal considerations we have cited such as the situation’s *op tempo* guiding the agent’s observation rate, and the need for situation prediction over multiple horizons accounting for both state and action dynamics, must be taken into account in order to properly assess *situation error*, a key step in the process model.

## Summary

There is a large literature on Situation Awareness and Situation Assessment that, to a large degree, treats the estimation of these states in isolation from many other functions needed to frame a complete, closed-loop process that not only estimates these states but addresses the overarching central issue for so many applications of situation control. Jakobson and a number of others, largely from the community of authors and attendees of the IEEE Cognitive Situation Management (CogSIMA) Conferences, have addressed many issues related to situation control and have tried to move the science forward by expanding the process view to a more holistic framework. This paper is a contribution to that collection of works, and also offers some limited remarks from the point of view of computational neurodynamics that is intended to lay the foundation for a dialog regarding the exploitation of Machine Intelligence within and central to the situation control paradigm. This is a complex space of thinking, of process architecting, of algorithmic design and development, and of human-machine interaction. As the broad technical communities of the world grapple with the development and exploitation of AI, ML, Machine Intelligence, and of the role of humans and of autonomous systems and behaviors, the need to frame the situation control process will be a central topic in the broadest sense; this paper is a small contribution to that goal.

## Author Contributions

Both authors listed have made a substantial, direct, and intellectual contribution to the work, and approved it for publication.

## Conflict of Interest

The authors declare that the research was conducted in the absence of any commercial or financial relationships that could be construed as a potential conflict of interest.

## Publisher’s Note

All claims expressed in this article are solely those of the authors and do not necessarily represent those of their affiliated organizations, or those of the publisher, the editors and the reviewers. Any product that may be evaluated in this article, or claim that may be made by its manufacturer, is not guaranteed or endorsed by the publisher.

## Footnotes

1 Sensemaking is not the same as understanding; sensemaking involves interplay between foraging for information and abstracting the information into a representation called a schema that will facilitate a decision or solution (http://www.peterpirolli.com/Professional/Blog__Making_Sense/Entries/2010/8/16_What_is_sensemaking.html).

2 Volume, velocity, variety, veracity, and value.

3
https://en.wikipedia.org/wiki/Gettier_problem

4
https://en.wikipedia.org/wiki/Kullback%E2%80%93Leibler_divergence

5
https://medium.com/rapids-ai/similarity-in-graphs-jaccard-vs.-the-overlap-coefficient-610e083b877d
